# Risk-driven or authority-based? Unraveling public policy compliance during the pandemic in China

**DOI:** 10.3389/fpubh.2025.1718039

**Published:** 2025-12-04

**Authors:** Hua Zhang, Chunyan Luo, Cheng Yang, Yanyan Ouyang, Xiuxian Deng

**Affiliations:** 1 School of Politics and Public Administration, Guangxi Minzu University, Nanning, China; 2 School of Public Policy and Management, Guangxi University, Nanning, China; 3 School of Government, Shenzhen University, Shenzhen, China

**Keywords:** authoritarian values, risk perception, government trust, professional trust, compliance behavior

## Abstract

**Background:**

Current research on pandemic compliance behavior primarily focuses on two motivational theories: normative and calculative motivations. Our study examines both by looking at public respect for authority and fear of infection risk to understand health policy compliance during COVID-19 in China.

**Methods:**

We conducted a survey with 2,305 Chinese citizens, using authority value and risk perception as independent variables, compliance behavior as the dependent variable, and government trust and professional trust as mediators. Structural equation modeling was employed to test the hypotheses.

**Results:**

Our study finds that, in terms of direct effects, both authoritarian values and risk perception are positively associated with compliance behavior among the Chinese public, with the effect of authoritarian values being stronger. Government and professional trust enhance the impact of authority value on compliance (trust enhancement) but weaken the impact of risk perception (trust paradox).

**Conclusion:**

From the perspective of normative and calculative motivations, compared with the calculative motivation based on individual risk perception, the normative motivation represented by authoritarian values demonstrates a stronger tendency toward policy cooperation, and the public is more likely to comply with public health policies when driven by this motivation. Trust in government and medical experts is crucial for health compliance behavior.

## 1 Introduction

In the face of emerging infectious diseases, the effectiveness of health policies depends not only on their scientific rigor and enforceability, but more fundamentally on the public’s willingness to cooperate with their implementation. During the global fight against COVID-19, governments around the world widely observed that even when policies were clearly articulated and technological resources were sufficient, public non-cooperation, distrust, or strategic compliance could still become major factors undermining disease control efforts (1, 2). Therefore, understanding why the public complies with public health policies has become a central issue of interdisciplinary concern and a critical component in managing public health crises. As noted by the WHO, understanding behavioral motivations and social environmental factors is one of the key pathways to achieving effective health governance and infectious disease control (3).

There are two primary motivations for studying compliance with individual health policies. Normative motivation emphasizes adherence driven by internalized values. Higher value identification helps individuals build a sense of obedience and belonging to authority, considering themselves part of the “ingroup” (4), which fosters more stable and higher levels of cooperative behavior. Under this psychological drive, individuals are more likely to actively respond to and comply with public health policies, showing respect for social norms and collective interests. Calculative motivation, on the other hand, highlights rational, risk-based compliance behavior (5). This approach reflects the individuals understanding of the necessity and urgency of policies based on personal perception, where individuals’ risk assessments of the health crisis directly influence their policy response. In addition to these two motivation theories, the Health Belief Model suggests that factors like beliefs, susceptibility, and self-efficacy influence attitudes toward health behaviors (6). These theories provide explanatory models for understanding individual actions during crises and help explain why people adopt specific preventive measures.

Existing research generally explores public compliance with health policies through two main pathways: normative motivation and calculative motivation. In-depth analyses have been conducted around various key variables, such as normative factors including values and beliefs (7), social norms (8, 9), social trust (10), and self-identity (11). As well as calculative factors such as risk perception (12, 13), needs and benefits (14), policy strictness and enforcement (15), and personal experience (16). However, most studies tend to focus on a single pathway when examining factors influencing public policy compliance, with relatively few studies comparing and investigating the relative mechanisms of normative and calculative motivations.

In recent years, some scholars have begun to integrate normative and calculative motivations into a unified analytical framework to provide a more comprehensive explanation of public responses to policy. For example, some studies have combined normative and calculative motivations to analyze the drivers of policy compliance among young Chinese elites (17). Other studies have focused on normative motivations such as value identification and social norms to examine the driving factors behind citizens’ policy compliance (18). Although these studies have made valuable theoretical and methodological contributions, research that simultaneously examines how both motivational pathways influence public behavior during large-scale public health events remains limited. In particular, after the pandemic entered a phase of normalized management, there was a shift in public attitudes and behaviors toward health policies. Compared to the early stage of the pandemic, when public responses were characterized by reactive compliance driven by risk avoidance under conditions of high uncertainty and perceived threat, the later stage reflected a shift toward more autonomous, value-based decision-making (1, 2), under normalized management, public behavioral decisions have increasingly reflected proactive choices based on personal value judgments, internal cognitive processes, and identification with social norms (19).

Therefore, this study takes authoritarian values under normative motivation and risk perception under calculative motivation as the main independent variables, and incorporates trust in government and professional institutions as mediating variables to construct a dual-path analytical model. It examines the mediating role of trust in both the normative and calculative motivation pathways and compares the distinct influences of these two pathways on policy compliance in the context of a public health crisis. In the comparison of normative and calculative motivational mechanisms, trust is often regarded as a key psychological mechanism that links individual motivations to policy response behaviors. On the one hand, the credibility of the government or professional institutions often affects individuals’ acceptance of authority, thereby enhancing the influence of normative motivation (10). On the other hand, during the risk assessment process, trust can reduce individuals’ perceived uncertainty regarding policy information, thereby influencing their rational judgments and behavioral decisions (1). Accordingly, we argue that trust may serve as a mediator in both motivational pathways, helping to uncover the psychological mechanism by which motivations are translated into compliance behavior. By comparing the different motivational pathways and their associated trust mechanisms, we aim to address ongoing debates in the literature, explain the variation in individuals’ policy compliance under different motivations, and deepen theoretical understanding of compliance behavior.

## 2 Literature review

### 2.1 Authoritarian values and compliance behavior under normative motivation

Explaining public health policy compliance through normative motivation suggests that individual behavior is guided by social norms, perceptions of fairness and reasonableness, and a sense of moral duty (19–23). Schwartz’s (24) Norm Activation Theory posits that individuals act based on personal norms or social obligations, with an emphasis on awareness of consequences and a sense of responsibility, which activates personal norms and influences behavior. Internalized normative attitudes include reverence and absolute adherence to social norms (25) and value identification (26). Previous research has shown that norm internalization positively impacts pro-environmental behavior (27), reduces smoking in public places (28), and promotes social distancing during the COVID-19 pandemic (29). Thus, normative motivation advocates enhancing public recognition of authority and policy legitimacy through perceived compliance with authority and contextual elements, thereby strengthening target group compliance behavior.

#### 2.1.1 Authoritarian values and individual compliance behavior

Studying public compliance behavior through normative motivations, those who value authority are individuals who typically adhere to established authorities, perceiving legitimate authority as a primary motivator for compliance. Some studies have found that trust in authority and a sense of fairness are significant variables influencing individual behaviors and attitudes, particularly during public crises (30). Researchers have noted that individuals with high authority values exhibit higher levels of social cooperation and positive civic behaviors, such as greater adherence to laws when there is perceived higher legal authority (31). Authority identification is rooted in prioritizing the protection of societal public interests, with value alignment influencing protective behaviors and attitudes (32), which contributes to public policy compliance to some extent (33). Previous studies indicate that authority values influence individual attitudes towards authoritative institutions and support for public policies (34), and may manifest behaviorally in compliance with tax payments, electoral participation, and other civic activities. Governments as policy implementers, whether stringent or facilitative, play a crucial role in promoting individual policy compliance (35). This underscores that stronger authority values in individuals correlate with higher willingness to comply, particularly evident in behaviors like mask-wearing and handwashing, especially pronounced during public health crises.

### 2.2 Risk perception and compliance behavior under calculative motivation

Explaining public health policy compliance through calculative motivation involves the rational analysis of the costs and benefits of cooperative behavior (5). This approach uses deterrence theory, protection motivation theory, and prospect theory. Deterrence theory emphasizes that individuals make rational decisions based on cost–benefit analysis (36), using legal deterrents and administrative penalties to prevent non-compliance, driven by fear of punishment. Protection motivation theory suggests that individuals’ decisions are based on balancing perceived external risks and their ability to avoid those risks (37). For instance, studies have found that high-risk perception among college students enhances their preventive behaviors against COVID-19 (13). Prospect theory posits that individuals often rely on heuristics to make quick judgments and decisions in uncertain situations (38). Therefore, in crisis situations with limited information, decisions are influenced by “irrational” factors and cognitive biases, and the final choices may not be the most rational ones.

#### 2.2.1 Risk perception and individual compliance behavior

During a health crisis, the public determines their behavioral intentions and response patterns based on their perception of risk factors (severity, susceptibility, intrinsic and extrinsic rewards) and their ability to avoid risks. Studying public compliance through the lens of risk perception under calculative motivation, risk perception is defined as an individual’s subjective assessment of potential threats (39). This perception plays a crucial role in high compliance behavior, driving individuals to take preventive measures to mitigate or avoid risks. During pandemics, research shows a significant positive correlation between risk perception and proactive crisis responses (12) and a significant negative correlation with passive responses (40). According to protection motivation theory, risk perception triggers threat and coping appraisals, leading to behavior change. This guides the individual to comply with health policies such as vaccination, social distancing, hand hygiene, and mask-wearing (41, 42). Compared to those with lower risk perception, individuals with higher risk perception reduced interpersonal interactions during the pandemic, confirming that risk perception significantly influences risk-related behaviors (43).

However, some studies show no significant relationship between risk perception and compliance behavior. High risk perception does not always lead to responsive behavior. Even with experience and high risk perception, individuals often fail to take appropriate actions (44), a phenomenon known as the “risk paradox.” This can be influenced by personal motivation, trust, sense of responsibility, and individual capability (45). In complex situations with multiple risks, high uncertainty may lead to feelings of helplessness, which in turn reduces the willingness to act. Thus, public compliance behavior can vary depending on the specific risk scenario.

### 2.3 The mediating role of trust

#### 2.3.1 Government trust and individual compliance behavior

Government trust encompasses confidence in the consistency of government actions and its fulfillment of promises. During pandemics, Chinese public trust in the government and social cohesion are notably strong (46). Research shows a positive correlation between government trust and adherence to preventive behaviors (47). The more people trust the government, the more they cooperate with its actions, such as following pandemic control measures (48). Conversely, low trust in the government leads to a lack of support for its policies (49). Many studies discuss government credibility extensively. Some scholars have pointed out that public acceptance of authority values strengthens trust in the central government (50). Authoritarian-oriented citizens show a significant positive correlation with political trust in East Asian societies (51), indicating that authority values enhance trust and confidence in political institutions (52). Trust in government decision-makers and policies can motivate individual compliance and foster more cooperative crisis behaviors (31).

Research indicates that individual trust in the government can reduce perceived risk during crises. Increased trust in official institutions leads to a decrease in negative perceptions of specific risks (53). In other words, the level of individual risk perception is directly influenced by trust in the government, which affects their coping strategies. Studies on the Ebola outbreak found that individuals who distrusted government control measures were less likely to take preventive actions, even when aware of the virus’s high risk and transmissibility (54, 55), and were more likely to engage in passive responses (56). Moreover, Political trust has been found to mediate the relationship between citizens’ value orientations or perceptions of institutional legitimacy and their willingness to comply with policies or cooperate with authorities (34). Accordingly, this study incorporates trust in government into the analytical framework to examine its mediating role in the process by which normative and calculative motivations influence policy compliance behavior.

#### 2.3.2 Professional trust and individual compliance behavior

Professional trust is based on confidence in medical experts and health institutions (34). During pandemics, professional trust is crucial for promoting public compliance. Trust in medical experts enhances individuals’ ability and sense of responsibility to address risks, leading to increased adoption of preventive health behaviors (57). During the 2009 H1N1 outbreak, health authorities’ recommendations for handwashing and mask-wearing led over one-third of the public to increase handwashing frequency (58). Those who trusted health authorities were more likely to follow recommended actions to control H1N1 (59). Trust in health institutions and experts, when aligned with public needs and values, serves as a valuable indicator of compliance (10, 60). Individuals with high authority identification tend to trust and follow health information from doctors and institutions (61, 62).

From a theoretical perspective, professional trust may serve as a critical psychological conduit between motivation and behavior. Within the normative motivation pathway, professional trust manifests as value-based identification with expert opinions, forming the psychological foundation for the internalization of social norms and adherence to professional authority. In the calculative motivation pathway, professional trust can reduce individuals’ skepticism and perceived uncertainty regarding health information, thereby contributing to the construction of a more stable risk assessment framework, which in turn facilitates more consistent decision-making responses (63). A trust gap between the public and health authorities can negatively impact compliance, as distrustful individuals are less likely to follow protective measures (64, 65). It follows that professional trust is not only a prerequisite for individuals to accept expert advice and health information, but also an essential mediating mechanism that connects individual motivations, whether based on normative value identification or rational risk assessment, to behavioral outcomes. In the dual-path theoretical framework proposed in this study, professional trust clarifies the psychological processes through which normative and calculative motivations are converted into policy compliance behavior.

### 2.4 Research framework and hypotheses

Based on the above research, we propose a model for how authority values and risk perception influence public compliance behavior through trust during public health crises. Previous studies have shown that public beliefs and authority compliance affect adherence to behavior (30). Recognizing the legitimacy of policy content and implementation promotes voluntary compliance. Authority values can enhance compliance with public health policies, suggesting that individual authority values increase willingness to cooperate with policies, leading to our hypothesis H1. Higher risk perception significantly improves compliance with crisis response and increases adherence to recommended behaviors (12), thus leading to our Hypothesis H2.

*H1*: The stronger an individual’s authoritarian values, the higher their level of compliance with public health policies.

*H2*: The higher the level of risk perception, the higher an individual’s compliance with public health policies.

Existing studies have shown that authoritarian values can not only directly shape individuals’ tendency to comply, but also indirectly promote compliant behavior by enhancing trust in political authority and professional institutions (66, 67). In the context of a public crisis, political trust and professional trust serve distinct psychological functions—representing perceived institutional legitimacy and epistemic credibility, respectively. These forms of trust act as important reference points for the public in making policy judgments and behavioral decisions. The former offers legitimacy for social order and collective goals, while the latter ensures the scientific and rational basis for risk response. The role of authoritarian values in activating both forms of trust constitutes a key mechanism through which normative motivations are transformed into policy compliance behavior. Based on this, we propose Hypothesis H3a and H3b.

*H3.a*: Authoritarian values enhance individual compliance behaviors through increased trust in the government.

*H3.b*: Authoritarian values enhance individual compliance behaviors through increased trust in professionals.

Trust is a key factor influencing risk perception. When individuals have lower risk awareness, trust becomes more critical. During public health events, the public evaluates information based on their general trust in social institutions (44). When risk perception is low, the role of trust becomes more prominent: individuals are more likely to rely on their general beliefs about institutions to assess the situation, rather than engage in active information seeking or independent judgment. In such cases, trust not only serves a cognitive substitute function, but also significantly influences individuals’ motivational basis for action. However, studies have also pointed out that high levels of trust may, in certain contexts, diminish individuals’ behavioral proactiveness. When the public places excessive trust in the government’s or experts’ ability to manage risks, they may underestimate their own role in the protection process, thereby reducing their willingness to engage in personal protective behaviors (45). In other words, when individuals delegate the responsibility of risk response to institutional or expert systems, risk perception may operate through the pathway of trust, thereby indirectly influencing their policy compliance tendencies. Accordingly, we propose Hypotheses H4.a and H4.b.

*H4.a*: Risk perception decreases individual compliance behavior through trust in government.

*H4.b*: Risk perception decreases individual compliance behavior through trust in professionals.

**Figure 1:**
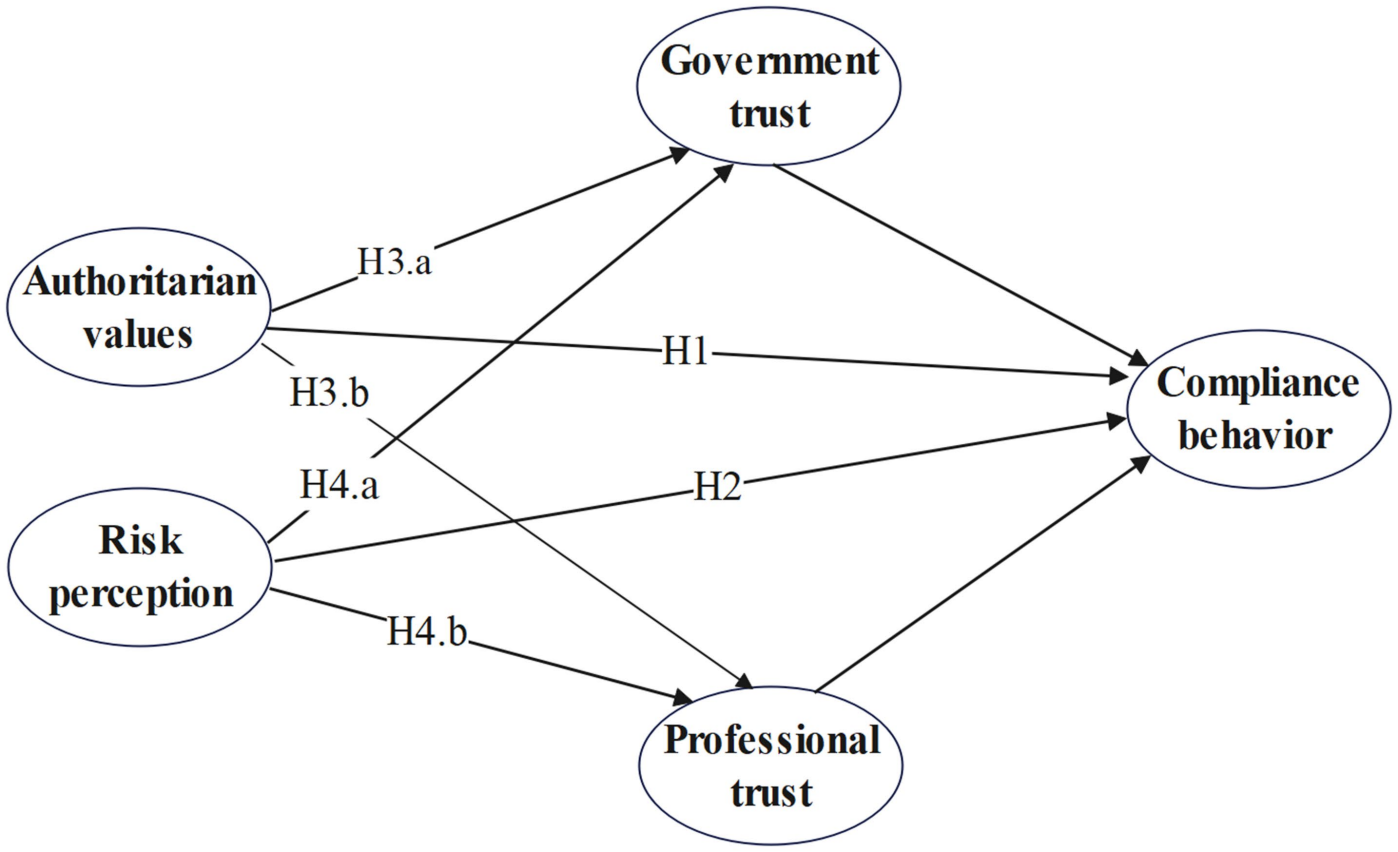
Research model.

In a public health crisis, trust affects individual compliance behavior through two mediating pathways (Figure 1). Authoritarian values may influence individual compliance behavior through the mediating roles of trust in government and professionals. Similarly, risk perception may affect individual compliance behavior through the mediating roles of trust in government and professionals.

## 3 Data and methods

### 3.1 Data collection

During the data collection phase, we distributed the survey link via social media platforms (e.g., WeChat) and invited participants to complete the questionnaire using an online snowball sampling method. Participants were required to voluntarily take part and complete an electronic informed consent process before proceeding with the survey. Given the use of snowball sampling and voluntary participation, younger individuals under the age of 30, who are more active on social media and more willing to engage, were overrepresented in the sample. As a result, respondents under the age of 30 accounted for as much as 98.6% of the collected survey sample.

Although the sample exhibits a certain degree of age-related bias, focusing on individuals under the age of 30 still holds important research value. Young people, particularly university graduates and current students, generally have a higher level of education, and tend to show greater willingness to express themselves and heightened sensitivity in areas such as political cognition, value judgment, and information processing. They are a core group in discussions of public opinion and ideology, with elite discourse playing a critical role in the formation of their value systems (68). Therefore, although the sample in this study is concentrated on young people, it remains both representative and insightful for examining the cognitive distribution and social attitudes toward current public issues. In total, we collected 2,506 survey responses, and, after screening out 201 invalid responses (e.g., logical inconsistencies or substantial missing data), we obtained 2,305 valid responses, resulting in a valid response rate of 91.98%.

### 3.2 Statistical methods

After data collection, we used STATA 16.0 for statistical analysis. Structural Equation Modeling (SEM) is an analytical approach that integrates measurement and structural models, allowing for the simultaneous estimation of relationships among multiple latent variables. It combines aspects of factor analysis and multiple regression (69). This study aims to employ SEM to test the mediating roles of government trust and professional trust in public policy compliance, as well as to examine the relational pathways between normative and calculative motivations. Prior to conducting SEM analysis, a Confirmatory Factor Analysis (CFA) was performed to assess the reliability and validity of the latent constructs in the measurement model. The analysis showed that the standardized factor loadings for all latent variables ranged from 0.71 to 0.97 and were statistically significant, indicating strong explanatory power of the observed items for their corresponding latent constructs. Cronbach’s α coefficients ranged from 0.70 to 0.94, and composite reliability (CR) values were between 0.70 and 0.94, all exceeding the commonly accepted threshold of 0.70, indicating good internal consistency of the scales. Additionally, the Average Variance Extracted (AVE) values all met or approximated the standard cutoff of 0.50, suggesting strong convergent validity of the latent variables. Overall, the reliability and validity indicators of the measurement model were satisfactory, making it suitable for subsequent structural model analysis. It should be noted that, due to the cross-sectional nature of the data, the SEM results reflect theoretical association paths among variables rather than causal relationships.

### 3.3 Variable and measurement

#### 3.3.1 Compliance behavior

Compliance behavior, the dependent variable in this study, refers to the individual alignment with and adjustment of their actions according to public policy regulations (68). During an outbreak, it specifically relates to adherence to public health policies, such as mask-wearing, hand hygiene, social distancing, and vaccination. Measures for identifying infected individuals also include big data screening policies, such as providing nucleic acid test results and staying at home during lockdowns (70). We used a 5-point Likert scale (1 = never compliant, 5 = always compliant) to survey respondents on their compliance with six behaviors: mask-wearing, hand hygiene, social distancing, vaccination, nucleic acid testing, and home isolation during lockdowns. Higher scores indicate greater adherence to public policies (Cronbach’s alpha = 0.824).

#### 3.3.2 Authoritarian values

Authority values reflect the public’s recognition and support for authority (71). This study measures authority values using a 5-point Likert scale (1 = strongly disagree, 5 = strongly agree) with two items: (1) “Trust in decision-makers such as government officials” and (2) “Government policies are generally correct.” Higher scores indicate stronger authority values (Cronbach’s alpha = 0.846).

#### 3.3.3 Risk perception

Risk communication in this study comprises four components. Following Fadel et al. (16), we used a 5-point Likert scale (1 = not worried at all, 5 = very worried) to measure respondents’ concerns about risk perception. Respondents rated their level of concern about the following statements: (1) “Fear of contracting the disease for themselves or their family”; (2) “Worries about lack of material security”; (3) “Concerns about employment issues during a public health crisis”; (4) “Worries about lockdowns or quarantines.” Higher scores indicate greater perceived risk (Cronbach’s alpha = 0.860).

#### 3.3.4 Government trust

Government trust is one of the mediating variables in this study. Based on research by Wachinger (44) and Clark (47), we used a 5-point Likert scale (1 = very dissatisfied, 5 = very satisfied) to measure respondents’ trust in the government. Respondents rated their trust with two statements: (1) “How trust are you with the central government?”; (2) “How trust are you with your local or county government?” Higher scores indicate greater trust in the government (Cronbach’s alpha = 0.859).

#### 3.3.5 Professional trust

Professional trust is another mediating variable in this study. To measure professional trust based on the level of trust in public health authorities (67), we used a 5-point Likert scale (1 = very distrustful, 5 = very trusting). Respondents rated their trust in two statements: (1) “How much do you trust healthcare professionals?”; (2) “How much do you trust health institutions?” Higher scores indicate greater professional trust (Cronbach’s alpha = 0.937).

#### 3.3.6 Demographic variables

Previous research indicates that individual characteristics significantly affect compliance behavior during a pandemic crisis (17). Therefore, this study includes demographic factors—such as gender, marital status, education, political affiliation, and residency—as control variables.

## 4 Results

### 4.1 Descriptive statistics

The demographic characteristics and means of the sample are shown in Table 1. Of the 2,305 respondents surveyed, 28.16% (649) were male and 71.84% (1,656) were female. Married individuals made up 5.2% (125), and those with a bachelor’s degree or higher constituted 58.83% (1,356). Most respondents 94.97% (2,189) were non-Communist Party members, while 5.03% (116) were Communist Party members. Urban residents represented 33.54% (773), and rural residents constituted 66.46% (1,532).

**Table 1:** Sample descriptive statistics (*N* = 2,305).

Variable		*N* (%)	Mean (SD)	Min/Max
Age	≤18	498 (21.61%)		
	19–29	1774 (76.96%)		
	≥30	33 (1.43%)		
Gender	Male (0)	649 (28.16%)		
	Female (1)	1,656 (71.84%)		
Marital status	Married (0)	125 (5.42%)		
	Unmarried (1)	2,180 (94.58%)		
Educational	Associate degree and below (0)	949 (41.17%)		
	Bachelor’s degree and above (1)	1,356 (58.83%)		
Political landscape	Non CCP Member (0)	2,189 (94.97%)		
	CCP Member (1)	116 (5.03%)		
Registered residence	City (0)	773 (33.54%)		
	Rural (1)	1,532 (66.46%)		
Authoritarian values	Trust in decision-makers		4.15 (0.92)	1/5
	policies are generally correct		3.90 (0.99)	1/5
Risk perception	Fear of infection		3.24 (1.17)	1/5
	Worried about material life		3.02 (1.18)	1/5
	Worried about employment		3.40 (1.21)	1/5
	Worried about being quarantined		3.26 (1.24)	1/5
Government trust	Central government		4.12 (0.85)	1/5
	Local government		3.92 (0.88)	1/5
Professional trust	Medical experts		4.06 (0.75)	1/5
	Health institutions		4.04 (0.77)	1/5
Compliance behavior	wearing masks		4.39 (0.76)	1/5
	hand hygiene		4.41 (0.71)	1/5
	maintaining social distance		3.98 (0.95)	1/5
	vaccination		4.66 (0.66)	1/5
	nucleic acid testing		4.73 (0.56)	1/5
	home isolation		4.67 (0.62)	1/5

Individuals’ mean scores for authoritative value orientation exceed those for risk perception. The mean trust in the central government stands at 4.12, while the means for professional trust are above 4. The measures advocated by the government all had high average scores above 4.5. However, “maintaining social distance” had a lower average score of 3.98. This indicates that during the pandemic, the public generally adhered well to these preventive measures, reflecting the Chinese government’s preference for restrictive measures to protect the public. The high compliance suggests that the public views these measures as both effective and necessary, driven by concerns about the consequences of non-compliance.

### 4.2 Structural equation model results

**Figure 2:**
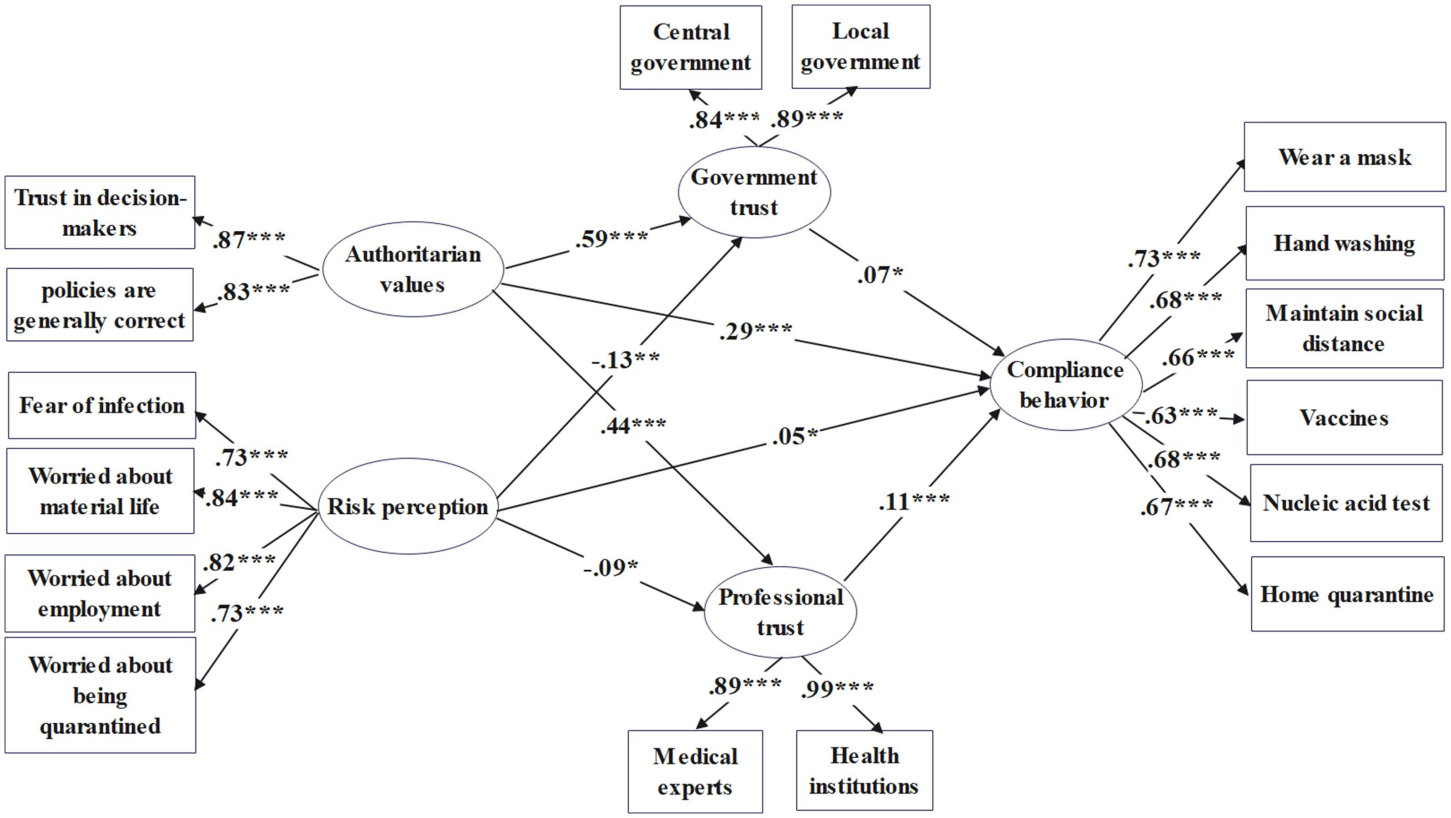
Structural equation modeling results. *p < 0.05; **p < 0.01; *** *p* < 0.001.

To further investigate the relationship between authoritarian values, risk perception, and compliance with public health policies, a structural equation model was employed to test the mediating effects of government trust and professional trust among authority values, risk perception, and individual compliance behaviors. The Structural equation model results, as depicted in Figure 2: Structural Equation Modeling Results, indicate good model fit with the following fit indices: X^2^ = 2009.765, DF = 126, CFI = 0.903, TLI = 0.887, RMSEA = 0.065, SRMR = 0.056. The comprehensive analysis suggests that the sample data fit the model well.

Analysis of direct effects is presented in Table 2. Controlling for demographic variables, authoritarian values significantly positively predict compliance behavior (a = 0.29, *p* < 0.001); risk perception also significantly positively predicts compliance behavior (a = 0.05, *p* = 0.021), confirming hypotheses H1 and H2. Authoritarian values positively predict government trust (a = 0.59, *p* < 0.001) and professional trust (a = 0.44, *p* < 0.001). Risk perception negatively predicts government trust (a = −0.13, *p* < 0.001) and professional trust (a = −0.09, *p* < 0.001). Government trust positively predicts compliance behavior (a = 0.07, *p* = 0.041), while professional trust positively predicts compliance behavior (a = 0.11, *p* < 0.001).

**Table 2:** SEM path parameters for influencing factors of compliance behavior.

Hypothesis	Estimate effects	S.E.	*p* value	Significance
Authoritarian values → Compliance behaviors	0.29	0.031	0.000	***
Risk perception → Compliance behaviors	0.05	0.024	0.021	*
Authoritarian values → Government trust	0.59	0.018	0.000	***
Authoritarian values → Professional trust	0.44	0.020	0.000	***
Risk perception → Government trust	−0.13	0.021	0.000	***
Risk perception → Professional trust	−0.09	0.021	0.000	***
Government trust → Compliance behaviors	0.07	0.033	0.041	*
Professional trust → Compliance behaviors	0.11	0.027	0.000	***
Female → Compliance behaviors	0.01	0.022	0.660	
Single→ Compliance behaviors	−0.05	0.021	0.018	*
College→ Compliance behaviors	0.13	0.022	0.000	***
CCP Member → Compliance behaviors	0.02	0.021	0.314	
Rural → Compliance behaviors	0.02	0.021	0.380	

*, **, *** indicate *p* < 0.05; *p* < 0.01; *p* < 0.001.

In terms of demographic variables, it was found that the public with a bachelor’s degree or higher education level exhibited greater compliance behavior (a = 0.13, *p* < 0.001); married individuals showed higher adherence (a = −0.05, *p* = 0.018). In contrast, gender (a = 0.01, *p* = 0.660), political affiliation (a = 0.02, *p* = 0.314), and household registration (a = 0.02, *p* = 0.380) did not significantly affect compliance behavior.

To further test the mediation mechanism, this study employed the bootstrap method proposed by Preacher and Hayes (72) to examine the indirect paths through government trust and professional trust between authoritarian values, risk perception, and public policy compliance behavior. The results, shown in Table 3. The results show that authoritarian values are indirectly associated with public policy compliance through government trust (β = 0.041, 95% CI [0.024, 0.061]), and also form a significant positive indirect path via professional trust (β = 0.038, 95% CI [0.025, 0.053]), thereby supporting Hypotheses H3.a and H3.b. In contrast, risk perception is negatively associated with compliance behavior through government trust (β = −0.015, 95% CI [−0.021, −0.008]) and professional trust (β = −0.009, 95% CI [−0.016, −0.004]), indicating significant negative indirect associations with public compliance behavior. These findings support Hypotheses H4.a and H4.b. A comparison of the standardized coefficients across different mediation paths reveals that in both sets of pathways, the mediation coefficients for government trust are higher than those for professional trust, suggesting that government trust plays a stronger mediating role within the model.

**Table 3:** Indirect impact analysis.

Hypothesis	Estimate effects	S. E.	Bootstrap95%	
			Lower limit	Upper limit
Authoritarian values → Government trust → Compliance behaviors	0.041	0.010	0.024	0.061
Authoritarian values → Professional trust → Compliance behaviors	0.038	0.007	0.025	0.053
Risk perception → Government trust → Compliance behaviors	−0.015	0.003	−0.021	−0.008
Risk perception → Professional trust → Compliance behaviors	−0.009	0.003	−0.016	−0.004

## 5 Discussion

Public compliance with health policies is a key factor influencing the effectiveness of central and local government responses during public emergencies. This study, grounded in the framework of normative and calculative motivations, explores the psychological mechanisms underlying individual policy compliance behavior. Based on the dimensions of authoritarian values and risk perception, a pathway model of public policy compliance behavior was constructed. Theoretical hypotheses were proposed regarding the mediating roles of government trust and professional trust.

### 5.1 Authoritarian values positively affects individual compliance behaviors

The results indicate that authority values have a significant positive effect on individual compliance behaviors, leading individuals to engage in preventive actions aligned with policy requirements. This finding supports previous research highlighting the role of authority recognition, personal values, and social norms in promoting compliance (31, 32, 47, 66). From a normative motivation perspective, authority values enhance individual support for health policies. Who respect authority are more likely to view government emergency measures as effective and experience fewer difficulties in complying with policies such as mask-wearing, social distancing, testing, and vaccination.

Importantly, the effect of authority values on individual compliance behavior is more pronounced even when sacrifices are required. Stronger authority values encourage higher compliance in social activities, such as travel restrictions and working from home. In China, young elites’ adherence to control measures is driven more by social responsibility and trust in the government rather than fear of infection or punishment (17). This demonstrates that individuals with strong authority values are more likely to proactively follow government guidelines to achieve collective societal goals and benefits during a pandemic.

### 5.2 Risk perception positively affects individual compliance behaviors

The survey results indicate that individuals with higher levels of risk perception are more inclined to wear masks, undergo nucleic acid testing, and receive vaccinations. This indicates that risk perception has a significant positive association with individuals’ protective behaviors, and the stronger the public’s perception of crisis risk, the more actively they engage in policy compliance behaviors. This finding is consistent with previous research (12, 43). Faced with sudden public health emergencies, people assess the extent to which their lives and property are at risk, considering the potential serious consequences, and thus tend to comply with emergency management policies. As public perception of risk increases, their adherence to current advocated preventive and protective behaviors also increases (42). During health crises, individuals may experience negative emotions, prompting more people to conform due to combined risk perception and conformity psychology when others adopt individual and public protective behaviors. This behavior serves to mitigate the pressure and fear brought about by risk situations.

### 5.3 Trust enhancement: authoritarian values enhance individual compliance behaviors through trust

This study reveals that during public health crises, citizen actions are closely related to their trust in the government, consistent with previous research (10, 73). The individual generally views expert systems and government agencies as legitimate authorities, forming the basis for their trust in crisis response measures. We found that individuals with stronger authority values tend to have greater trust in government effectiveness during pandemic control, indicating that authority values enhance public trust in the government (51, 52, 71). Trust facilitates individual cooperative behavior (74), This trust mechanism promotes individual compliance with public health policies.

Moreover, trust in health institutions and medical personnel also plays a role in encouraging public participation in health preventive behaviors (75). Professional trust, developed through ongoing interactions with healthcare providers, leads individuals with high trust to follow preventive guidance from doctors. Our findings indicate that authoritarian values are indirectly associated with public policy compliance through professional trust, suggesting that trust in professional institutions or expert systems serves as a key mechanism linking authoritarian attitudes to behavioral responses. In China, people generally show high trust in medical experts’ advice and align with their values, leading to cooperative behaviors. Thus, the internalization of authority values strengthens public trust in government and health institutions, which in turn promotes compliance with public health policies.

### 5.4 Trust paradox: risk perception weakens individual compliance behavior through trust

Trust, as a critical psychological factor, can mitigate negative risk perceptions and facilitate risk-taking through subjective trust in government and experts (53). However, our empirical findings indicate that government trust and professional trust attenuate the relationship between risk perceptions and individual compliance behaviors. Previous research suggests that individuals with low-risk perceptions are less likely to respond to warnings and adopt precautionary measures compared to those with high-risk perceptions (76). Our results indicate that trust plays a crucial role in the relationship between risk perception and policy compliance. Indeed, similar findings from previous studies indicate that high risk perceptions do not necessarily promote individual emergency preparedness, a phenomenon known as the “risk perception paradox” (44). Specifically, trust in government may lead to decreased risk perceptions, thereby reducing compliance with risk management measures (53, 77).

During pandemics, this phenomenon may be particularly pronounced. One explanation is that individuals’ heightened fear of infection may lead them to place more trust in government policies and medical experts, thereby reducing compliance with public health policies, known as the “support paradox” (73). Another explanation involves optimistic bias, where individuals with higher levels of social trust may adopt an optimistic view towards the pandemic, believing they are less susceptible to risks compared to others, and thus taking fewer precautionary measures (63, 78). Additionally, trusting in the government’s emergency response capabilities may lead individuals to reject restrictive compliance behaviors such as undergoing nucleic acid testing or refraining from going out during outbreaks. Moreover, based on the Trust, Confidence, and Cooperation (TCC) model (45), reducing risk-related worries not only helps enhance public trust in the government and healthcare institutions but may also increase individuals’ willingness to comply with public health policies. During major public health emergencies, normative and calculative motivations exhibit distinct mechanisms in driving individual behavior, necessitating a distinction in understanding the diverse motivational influences on public behavior to implement more targeted preventive measures.

### 5.5 Analysis of compliance behavior differences among different population groups

Analyzing behavioral differences among various groups during the pandemic, we found that married individuals generally showed stronger tendencies toward health policy compliance (47). These groups, typically more cautious and worried about the risks to their families after being infected with the virus. Individuals with a bachelor’s degree or higher education level are more likely to adopt preventive measures (16), due to better access to public health information and a tendency to trust scientific guidance. Gender, political landscape and Registered residence show no significant differences in compliance with public health policies, reflecting the universality of policies and the effectiveness of emergency systems in ensuring public adherence during pandemics. Therefore, beyond enhancing public health education and risk awareness, targeted development and promotion of preventive measures tailored to different groups’ characteristics and needs will be more effective.

## 6 Conclusion, policy implications, and limitations

This study finds that both normative and calculative motivations are significant drivers of public health compliance behavior. First, within the normative motivation pathway, the study finds that authoritarian values influence public compliance behavior indirectly through trust in government and experts. Specifically, when individuals endorse authoritarian values and hold higher levels of trust in government and experts, they are more likely to internalize policy requirements and demonstrate stronger compliance with public health policies. This finding clarifies the mediating role of trust in the transformation of authoritarian values into behavior, deepening theoretical understanding of how normative motivation functions. Second, within the calculative motivation pathway, the study reveals a theoretically significant result known as the trust paradox. While risk perception significantly and positively influences public compliance behavior, higher levels of trust in government and experts may reduce individuals’ perceived risk, which in turn diminishes their likelihood of compliance. In this context, trust serves a weakening function in the risk–compliance relationship, a phenomenon commonly referred to as the “trust paradox.” This study expands the theoretical boundaries of trust research, and offers a novel explanatory framework for understanding why low compliance may occur even in high-trust societies.

The findings of this study have significant policy implications. First, the results highlight the importance of both normative and calculative motivations in promoting compliance with health measures. When formulating and implementing public health policies, it is essential to strengthen the authority of government bodies for groups with strong authority values, thereby enhancing public identification with policy goals and encouraging compliance (26). For groups relying on risk perception, it is necessary to enhance risk education to improve their understanding of risks. Second, the study underscores the critical role of government and professional trust in public health emergency management. Enhancing the trustworthiness of government and health institutions not only boosts individual compliance but also improves overall pandemic response effectiveness. Finding ways to encourage voluntary compliance can save medical resources and, more importantly, protect individual health.

There are some limitations in our study. First, due to the use of convenience sampling, the survey sample was not sufficiently diverse and skewed younger, affecting the representativeness of the data and the generalizability of the conclusions. Second, the questionnaire was collected during the normalized phase of pandemic control in China, reflecting only the individual’s mindset at that time, policy compliance needs to be investigated at different stages of the pandemic. Therefore, the findings primarily reflect the public’s mindset at the time and possess a certain degree of temporal specificity. Third, this study only considered authority values under normative motivation and risk perception under calculative motivation. Future research should expand the scope of study subjects, compare changes in individual compliance motivations at different stages of development, and consider the interactive effects of more factors. Finally, as this study is based on a cross-sectional design, it allows for the examination of path associations among variables but cannot fully establish causal relationships. Future research is recommended to employ longitudinal data or experimental designs to gain deeper insights into the causal mechanisms among variables.

## Data Availability

The raw data supporting the conclusions of this article will be made available by the authors, without undue reservation.
